# IGSF11：肿瘤免疫治疗新靶点

**DOI:** 10.3779/j.issn.1009-3419.2025.106.14

**Published:** 2025-05-20

**Authors:** Zhibo FENG, Xiyang TANG, Yao LV, Zhaoxiang WANG, Zhixiang ZHANG, Longyan NIE, Shaohui RU, Jinbo ZHAO

**Affiliations:** ^1^710069 西安，西北大学医学院; ^1^School of Medicine, Northwest University, Xi'an 710069, China; ^2^710038 西安，空军军医大学唐都医院胸腔外科; ^2^Department of Thoracic Surgery, Tangdu Hospital, Air Force Medical University, Xi'an 710038, China; ^3^472099 三门峡，三门峡市中医院; ^3^Sanmenxia Hospital of Traditional Chinese Medicine, Sanmenxia 472099, China

**Keywords:** IGSF11, 免疫治疗, 免疫检查点, 肿瘤微环境, IGSF11, Immunotherapy, Immune checkpoint, Tumor microenvironment

## Abstract

免疫检查点阻断疗法在多种类型的肿瘤治疗中显示出显著的疗效，但其临床应用仍面临着如免疫应答率低及免疫相关不良事件的挑战。免疫球蛋白超家族成员11（identification of immunoglobulin superfamily 11, IGSF11）是一种抑制性免疫检查点分子，作为T细胞活化的V型免疫球蛋白结构域抑制因子（V-domain immunoglobulin suppressor of T cell activation, VISTA）的特异性配体，其通过IGSF11/VISTA轴抑制T细胞功能，有潜力成为免疫治疗肿瘤的新靶标。IGSF11广泛表达于多种恶性肿瘤中，其调控机制因肿瘤类型不同而存在差别。已有研究证明阻断IGSF11与VISTA结合或对IGSF11进行特异性抑制，可产生抗肿瘤作用。IGSF11与患者预后密切相关，但其在不同肿瘤中的预后价值不同。本文将对IGSF11的结构特征、表达调控机制、与VISTA的相互作用及其在肿瘤微环境中的作用进行系统概述。

## Abstract (en)

Immune checkpoint blockade therapy has demonstrated remarkable efficacy in treating various malignancies; however, its clinical application remains challenged by low response rates and immune-related adverse events. Immunoglobulin superfamily member 11 (IGSF11), an inhibitory immune checkpoint molecule, serves as a specific ligand for the V-domain immunoglobulin suppressor of T cell activation (VISTA). Through the IGSF11/VISTA axis, it suppresses T cell function and represents a promising novel target for cancer immunotherapy. IGSF11 is widely expressed across multiple tumor types, though its regulatory mechanisms vary depending on the malignancy. Studies have confirmed that blocking the IGSF11-VISTA interaction or specifically inhibiting IGSF11 exerts antitumor effects. While IGSF11 is closely associated with patient prognosis, its prognostic significance differs among cancer types. This review systematically summarizes the structural characteristics of IGSF11, its regulatory mechanisms, interaction with VISTA, and functional role within the tumor microenvironment.

肿瘤治疗模式经历了最初以外科手术、放疗和传统化疗为主，随后转向精准的分子靶向干预，直至发展为以免疫检查点抑制剂、过继性细胞疗法^[1]^为代表的免疫治疗模式的过程。免疫检查点阻断疗法通过逆转肿瘤微环境的免疫抑制状态，重新激活免疫效应细胞对恶性肿瘤的清除功能，从而实现抗肿瘤治疗的效果。

近些年，如帕博利珠单抗、伊匹木单抗等以程序性死亡受体1（programmed death receptor 1, PD-1）抑制剂和细胞毒性T淋巴细胞相关蛋白4（cytotoxic T-lymphocyte-associated protein 4, CTLA-4）抑制剂为代表的免疫检查点抑制剂，已在非小细胞肺癌、肝细胞癌、乳腺癌^[2-4]^等多种癌症治疗中取得显著的临床疗效。然而，一些患者仍面临免疫耐药问题，并出现包括甲状腺炎在内的免疫相关不良事件（immune-related adverse events, irAEs），这些因素限制了临床获益和应用范围^[5-8]^。为提高免疫治疗的效果、开发更安全有效的联合治疗策略，降低irAEs的发生率，亟需进行新型免疫检查点及其靶分子的发现与验证。

免疫球蛋白超家族成员11（identification of immunoglobulin superfamily 11, IGSF11）又称BT-IgSF、CT119、CXADRL1、VSIG3，其作为一种细胞黏附蛋白，属于由柯萨奇病毒-腺病毒受体（coxsackievirus and adenovirus receptor, CAR）、内皮细胞选择性黏附分子及CAR样膜蛋白组成的免疫球蛋白细胞黏附分子（immunoglobulin cell adhesion molecules, IgCAMs）亚群，以钙非依赖性方式介导细胞间同型黏附作用^[9]^。IGSF11在大脑、睾丸、破骨细胞和黑素细胞中特异性表达，在正常组织中起到增殖、黏附、迁移及突触形成等生理功能^[10-15]^。近年研究发现，IGSF11在多种恶性肿瘤（如乳腺癌、黑色素瘤、胶质瘤等）及部分肿瘤相关炎症细胞中表达且与肿瘤细胞增殖、转移及免疫调控相关。在皮肤黑色素瘤、胶质瘤、乳腺癌中，IGSF11特异性结合VISTA，从而抑制T细胞的免疫作用，减弱机体抗癌效果^[16-18]^。IGSF11在头颈部鳞状细胞癌中通过调控糖酵解途径影响肿瘤进展^[19]^。值得注意的是，虽然IGSF11已被证实参与多种恶性肿瘤的重要调控作用，但其在肺癌（如非小细胞肺癌）中的表达特征、促癌机制等方面的研究较为有限，需要进一步的实验探索和临床验证。本文将对IGSF11的分子结构特征、生物学功能及其在恶性肿瘤中的表达调控机制进行系统的综述，探讨其在肿瘤微环境中的免疫调控作用，为开发基于IGSF11靶点的肿瘤免疫治疗提供新思路及理论依据。

## 1 IGSF11的结构与特征

对IGSF11结构的研究可确定其功能性表位，为开发特异性靶向抗体或化合物提供重要的分子结构依据。*IGSF11*基因定位于人类3号染色体，通过选择性剪接产生两种结构高度保守但功能存在差异的异构体（IGSF11-1和IGSF11-2）。IGSF11-1作为II型跨膜蛋白缺乏N端信号肽，其胞内免疫球蛋白样结构域表明其可能参与细胞内信号转导及细胞骨架作用，但目前缺乏对此亚型的研究；而IGSF11-2作为I型跨膜蛋白含有N端信号肽，其胞外免疫球蛋白结构域与配体识别和细胞黏附相关，因此被视为主要功能亚型^[20]^。

IGSF11蛋白及其亚型结构的示意见图1。IGSF11的分子结构主要包含4个部分：N端信号肽序列、跨膜结构域、胞外结构域（由膜远端的IgV型结构域、膜近端的IgC2型结构域组成）和胞内结构域。其胞外结构域在大肠杆菌中以包涵体形式表达，并通过氢键相互作用形成功能性蛋白二聚体^[21]^。值得注意的是，IGSF11的C端含有PDZ（PDZ是3个含有该结构域的蛋白质PSD-95、Dlg和ZO-1的首字母缩写）结合基序，可与突触后致密区蛋白95（posy-synaptic density protein 95, PSD-95）、紧密连接蛋白-1（zonula occludens-1, ZO-1）等含PDZ结构域的支架蛋白相互作用。在Xie等^[21]^的研究中，通过X射线解析了IGSF11胞外结构域（IgV-IgC2）的高分辨率三维结构（2.64Å），并进一步证明了IGSF11与VISTA的特异性结合，为设计针对IGSF11/VISTA通路的抗体或靶向化合物提供了结构基础。

**图 1:**
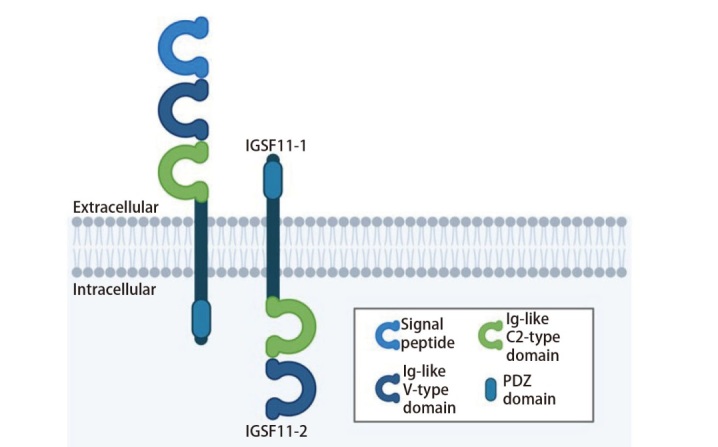
IGSF11蛋白及其亚型结构示意图

## 2 IGSF11的表达

### 2.1 IGSF11在正常组织中的表达

IGSF11在正常组织中的表达极低，主要表达于大脑和睾丸组织^[10]^中，在小脑、破骨细胞及其前体^[11]^和黑素细胞及其前体^[12]^中也有表达，其在卵巢、肾上腺、肾脏和甲状腺中表达水平较低^[17]^。

### 2.2 IGSF11在肿瘤组织中的表达

IGSF11在肿瘤组织以及部分肿瘤相关炎症细胞^[16]^中高表达，但其表达模式和临床意义在不同肿瘤类型中存在差异。现有的研究^[16-18,22,23]^发现IGSF11在结直肠癌、肝细胞癌、高级别胶质瘤、肠型胃癌、子宫内膜癌、黑色素瘤和乳腺癌中均呈现高表达。在乳腺癌中IGSF11表达水平与肿瘤细胞的侵袭性呈正相关，IGSF11不仅在肿瘤细胞中高表达，还在肿瘤免疫浸润细胞（如CD45^+^淋巴细胞和CD68^+^巨噬细胞）中显著富集^[18]^。值得注意的是，与其他类型的癌症相比，IGSF11在皮肤黑色素瘤中的表达水平最明显，但其表达水平与免疫细胞浸润无相关性，仅与肿瘤纯度呈正相关^[17]^。在胶质瘤中，IGSF11在多种免疫检查点（包括PD-1、CTLA-4等）中的表达最高。与低等级胶质瘤相比，高等级胶质瘤（III、IV级）的IGSF11 mRNA表达水平更高；免疫组化染色确定IGSF11蛋白在所有等级的胶质瘤肿瘤和炎症细胞中均显著表达^[16]^。

在不同肿瘤中IGSF11的表达与VISTA及其他免疫检查点如PD-1、细胞程序性死亡配体1（programmed cell death ligand 1, PD-L1）、CTLA-4等的表达存在不同的相关性。在胶质瘤中IGSF11的表达与PD-1和VISTA呈正相关，但与CTLA-4呈负相关^[16]^；然而，在胶质母细胞瘤中Yuan等^[24]^却发现IGSF11表达与VISTA呈负相关。在皮肤黑色素瘤中IGSF11表达与CTLA-4、PD-1和VISTA呈负相关^[17]^。

目前，关于IGSF11表达受到的调控机制研究仍较有限，其具体分子机制仍需进一步探索。在乳腺癌中，转化生长因子-β（transforming growth factor-β, TGF-β）诱导的上皮-间质转化途径可上调IGSF11的表达^[25]^；VISTA/IGSF11/PSGL-1轴中的分子存在相互影响关系，通过siRNA沉默VISTA/IGSF11/PSGL-1轴中的VISTA或血小板反应蛋白-1（P-selectin glycoprotein ligand-1, PSGL-1）会导致IGSF11 mRNA表达水平升高但蛋白水平下降，说明IGSF11表达可能通过补偿机制增强转录，同时存在转录后调控抑制翻译过程^[18]^。在肺癌中，miR-125a-5p可抑制IGSF11的表达，而RNA结合蛋白HuR能阻断腺苷甲基化miR-125a-5p与GW182的结合，减弱miR-125a-5p对IGSF11的抑制作用，导致IGSF11表达上调^[26]^。

## 3 IGSF11与VISTA的相互作用

目前研究^[27]^表明，VISTA是IGSF11唯一明确鉴定的配体。除VISTA外，其他潜在的配体作用仍停留在预测阶段，需要进一步的实验验证。虽然IGSF11可与PSD-95等支架蛋白相互作用，但尚无研究明确证实两者之间的直接结合作用。

IGSF11作为抑制性结合配体与B7家族成员VISTA特异性结合，但不直接作用于PD-1、CTLA-4等其他免疫检查点^[17,28]^。多项研究^[21,27,29]^通过酶联免疫吸附测定和免疫共沉淀实验证明了VISTA与IGSF11的直接结合作用。Xie等^[21]^进一步鉴定出IGSF11和VISTA关键结合界面的氨基酸残基，深入解析了其分子互作位点。在结构层面，Li等^[30]^基于实验验证的VISTA晶体结构与AlphaFold预测的IGSF11构象，构建出VISTA/IGSF11复合物的三维结构，不仅在结构层面证明了IGSF11与VISTA的特异性结合作用，还明确了多个界面关键残基（如His122、His123等）的空间分布，为靶向该通路的药物设计提供了关键结构依据。

IGSF11和VISTA的结合受多种因素调控。IGSF11与VISTA的结合具有剂量依赖性和可饱和性（4 μmol/L VISTA达到饱和）特性，且未标记的VISTA、IGSF11特异性抗体可以竞争性抑制两者的特异性结合^[28]^。此外，VISTA和IGSF11相互作用受酸碱环境调控。在生理条件（pH=7.4）下，VISTA对IGSF11的亲和力显著高于酸性条件（pH=6.0），提示酸化的肿瘤微环境可通过该通路影响免疫调节功能^[31]^。

## 4 IGSF11的调控作用

### 4.1 IGSF11在正常组织中的调控作用

IGSF11主要作为细胞黏附分子在正常组织中发挥作用，且被证明可调节缝隙连接蛋白的表达或定位^[10,32]^。IGSF11在神经系统中调节星形胶质细胞缝隙连接介导的细胞间通讯，参与神经元发育、突触形成和颗粒细胞的增殖和分化^[10,11,13,15]^；在睾丸组织中IGSF11通过调节连接蛋白43的定位维持血-睾屏障完整性及协调生殖细胞减数分裂进程，其功能异常可导致男性不育等症状^[32,33]^；在骨组织中IGSF11参与调控破骨细胞成熟和骨吸收^[34]^，通过抑制丙酮酸激酶同工酶M2（pyruvate kinase isozyme type M2, PKM2）等糖酵解关键酶活性，促进破骨细胞分化^[35,36]^。此外，IGSF11还参与黑素细胞定向迁移、黏附^[12,14]^等生物学过程。

IGSF11可与PSD-95、ZO-1等支架蛋白相互作用，从而能够在大脑、破骨细胞（通过与PSD-95结合^[15,34]^）和血-睾屏障（通过与ZO-1结合^[32]^）中形成蛋白复合体，调控神经系统、骨骼和男性生殖系统的生理功能。

### 4.2 IGSF11在肿瘤组织中的调控作用

IGSF11调控肿瘤细胞的增殖、转移、凋亡及细胞周期等表型。敲低*IGSF11*基因可以抑制胃癌细胞的增殖和活性^[22]^。在皮肤黑色素瘤中敲低IGSF11不仅抑制肿瘤细胞的增殖、转移，还可以促进细胞凋亡，但不影响细胞周期分布。在乳腺癌中TGF-β诱导上皮-间质转化，上调lincRNA Platr18表达，促进IGSF11的表达，通过促进轴突形成以支持肿瘤转移^[25]^。头颈部鳞状癌的研究^[19]^也证实*IGSF11*基因敲低后，肿瘤细胞的活力、转移、侵袭及克隆形成能力均降低。

IGSF11作为重要的免疫调节分子，与VISTA特异性结合发挥免疫抑制作用，影响机体免疫调控，促进实体瘤恶性进展。IGSF11发挥类似抑制性免疫检查点分子（如PD-1/PD-L1轴）的作用介导免疫逃逸，抑制T细胞增殖，减少细胞因子和趋化因子产生。Wang等^[27]^发现IGSF11不仅抑制T细胞活化，还以剂量依赖性方式显著减少了外周血单个核细胞（peripheral blood mononuclear cells, PBMCs）和T细胞中促炎细胞因子如白细胞介素17（interleukin-17, IL-17）和趋化因子的分泌，趋化因子包括C-C基序趋化因子3[chemokine (C-C motif) ligand 3, CCL3]、CXC基序趋化因子11[chemokine (C-X-C motif) ligand 11, CXCL11]，导致免疫细胞（单核细胞、树突状细胞及肿瘤相关巨噬细胞）在肿瘤微环境中的浸润减少，从而形成免疫抑制环境。这些结果表明IGSF11/VISTA通路发挥着负性调控T细胞功能的作用。Shekari团队^[17]^发现沉默IGSF11可显著抑制黑色素瘤的恶性进展，改变了T细胞细胞因子的表达，并诱导T细胞向促炎表型转化，表现为促炎细胞因子[干扰素-γ（interferon-γ, IFN-γ）和IL-12]表达上调和抗炎细胞因子[IL-10、TGF-β和肿瘤坏死因子-α（tumor necrosis factor-α, TNF-α）]表达下降。在肺癌中IGSF11能显著抑制IL-2激活的PBMC对肺癌细胞（H1975）的细胞毒性作用，使用抗IGSF11抗体可恢复PBMC介导的细胞溶解活性，证实IGSF11是调控肺癌细胞免疫逃逸的关键分子^[26]^。高表达IGSF11的胶质瘤患者虽表现出显著的CD4^+ ^T细胞和CD8^+ ^T细胞浸润，但其通过上调TGF-β等免疫抑制因子抑制T细胞功能，表现出有限的免疫效能。此外，IGSF11在胶质瘤表面和浸润的炎症细胞上分别作为配体和受体表达，可增强IGSF11与VISTA的结合及免疫抑制作用^[16]^。值得注意的是，IGSF11可能通过调控代谢途径影响肿瘤生长。在头颈鳞状细胞癌中，Nie等^[19]^发现IGSF11作为风险基因与糖酵解过程相关。通过Western blot检测显示，敲低*IGSF11*基因后，糖酵解关键酶葡萄糖磷酸果糖激酶（phosphofructokinase, PKFP）和PKM2的蛋白表达降低。Liu等^[37]^也揭示了IGSF11作为糖酵解免疫相关基因与口腔鳞状细胞癌患者的预后相关性。然而目前对于IGSF11在糖酵解代谢途径中的调控机制研究较为有限，未来有待深入研究肿瘤相关代谢机制。

## 5 靶向IGSF11/VISTA通路药物的研究进展

近年来，靶向VISTA/IGSF11通路的药物研发已经取得了显著进展，主要包括两大类：IGSF11靶向药物（如小分子抑制剂K284-3046和多肽疫苗IGSF11-9V/9L）以及抗VISTA抗体（VSTB112、BMS767、SG7、HMBD-002和KVA12123）。这些药物通过特异性阻断IGSF11/VISTA相互作用，有效逆转免疫抑制微环境，增强抗肿瘤免疫应答，达到抑制肿瘤恶性进展的作用。IGSF11及VISTA的相关靶向药物的总结见表1^[21,22,31,38-40]^。

**表 1:** IGSF11及VISTA的靶向药物进展

Target	Drug type	Drug name	Mechanism of action	Effect	Reference
IGSF11	Small molecule inhibitor	K284-3046	Inhibits the function of IGSF11 protein on T cells	Enhances PBMC activity and sustains PBMC proliferation	^[21]^
IGSF11	Peptide vaccine	IGSF11-9V, IGSF11-9L	Binds to HLA-A*02:01 epitopes	Enhances cytotoxic T lymphocyte functionality	^[22]^
VISTA	Monoclonal antibodies	VSTB112	Blocks the interaction between VISTA and IGSF11	Restores T cell activity	^[31,38]^
VISTA	Monoclonal antibodies	BMS767	Blocks the interaction between VISTA and IGSF11	Restores T cell activity	^[31]^
VISTA	Monoclonal antibodies	SG7	Blocks the interaction between VISTA and IGSF11	Reduces myeloid-derived suppressor cells and increases CD4^+^ and CD8^+^ T cell populations	^[31]^
VISTA	Monoclonal antibodies	HMBD-002	Blocks the interaction between VISTA and IGSF11	Reduces IFN-γ secretion, promotes pro-inflammatory Th1 cell responses, and enhances immune cell infiltration	^[39]^
VISTA	Monoclonal antibodies	KVA12123	Blocks the interaction between VISTA and IGSF11	Induces pro-inflammatory phenotypes and recruits/activates T cells and NK cells via Fc-dependent mechanisms	^[40]^

VISTA: V-domain immunoglobulin suppressor of T cell activation; HLA-A*02:01: human leukocyte antigen-A*02:01; PBMC: peripheral blood mononuclear cell; IFN-γ: interferon-γ; Th1: T helper 1 cell; NK: natural killer.

### 5.1 IGSF11靶向药物研究

#### 5.1.1 K284-3046

Xie等^[21]^基于VISTA/IGSF11蛋白质互作模型，发现了一种IGSF11小分子抑制剂K284-3046，K284-3046能结合于IGSF11的PRO46、SER48等8个氨基酸位点，发挥双重调控作用。一方面减弱了IGSF11对活化PBMCs的免疫抑制作用，增强抗肿瘤活性；另一方面提高了IGSF11对PBMCs增殖的抑制作用，避免过度的炎症反应。

#### 5.1.2 IGSF11-9V和IGSF11-9L

在胃癌中基于IGSF11设计的多肽疫苗可以有效增强细胞毒性T淋巴细胞（cytotoxic T lymphocytes, CTLs）的功能。Watanabe等^[22]^合成了IGSF11的两个肽序列（IGSF11-9V和IGSF11-9L）用于胃癌的免疫治疗，其以人类白细胞抗原-A*0201（human leukocyte antigen-A*02:01, HLA-A*02:01）限制性方式诱导CTLs对高表达IGSF11的胃癌细胞产生细胞毒性。

### 5.2 抗VISTA抗体靶向药物研究

#### 5.2.1 VSTB112

VSTB112作为靶向VISTA的IgG1-κ抗体，通过特异性结合VISTA的C-C′环（H121和H122残基）及相邻螺旋区域，在体外抑制VISTA与IGSF11的相互作用^[31]^。在前几年进行的I期临床试验^[38]^期中评估了VSTB112在晚期肿瘤（包括肺癌、胰腺癌、头颈部癌、结直肠癌和宫颈癌）中的安全性、耐受性和药代动力学特征，但因未知原因提前终止试验，且其后续临床开发前景尚不明确。

#### 5.2.2 BMS767

BMS767作为一种靶向VISTA的抗体，能特异性识别VISTA蛋白前C-C'环区域的组氨酸残基（H121、H122）及临近残基。BMS767可以有效阻断IGSF11与VISTA的相互作用，且表现出与独特的pH依赖性结合特征^[31]^。

#### 5.2.3 SG7

SG7与BMS767、VSTB112、PSGL-1及IGSF11蛋白都与VISTA的H122残基结合，因此均可以有效阻断PSGL-1和IGSF11与VISTA的相互作用。SG7与VISTA的亲和力比BMS767或VSTB112高25-50倍，且能竞争性抑制其他抗体（VSTB112和BMS767）与VISTA的结合，阻断VISTA介导的T细胞抑制功能。此外，SG7与IGSF11的结合不受pH影响。临床前研究^[31]^显示，在黑色素瘤、乳腺癌小鼠模型中，SG7单药治疗可有效抑制肿瘤生长，且增加了乳腺癌（4T1细胞）模型中免疫细胞的数量，在结肠癌（MC38细胞）模型中，SG7联合抗PD-1治疗也展现出协同抗肿瘤的结果。

#### 5.2.4 HMBD-002

HMBD-002是一种非消耗性IgG4抗VISTA抗体，主要与VISTA的C-C'环结合，并以剂量依赖性方式拮抗VISTA/IGSF11作用，抑制IFN-γ的分泌。HMBD-002重塑了肿瘤免疫微环境，诱导免疫环境向促炎表型的转变，增加免疫细胞（CD11b^+^巨噬细胞、CD8^+ ^T细胞）的数量，并增强肿瘤浸润白细胞的特异性细胞毒作用。在结直肠癌、肺癌和乳腺癌小鼠模型中，HMBD-002可有效抑制肿瘤生长^[39]^。

#### 5.2.5 KVA12123

KVA12123是一种抗VISTA的IgG1-κ单克隆抗体，在中性和酸性pH下与VISTA结合，阻断与VISTA相互作用的配体（如IGSF11）。其通过激活髓系细胞，将肿瘤微环境重塑为促炎表型，并以Fc依赖性方式募集和激活T细胞与NK细胞。在结肠癌和黑色素瘤模型中，KVA12123单药治疗或与PD-L1抑制剂联合使用时展现出强大的抑制肿瘤生长的能力^[40]^。

## 6 预后预测

IGSF11在不同肿瘤的预后中表现出不同的特性。高表达IGSF11与胶质瘤、头颈部鳞状细胞癌和胃癌的不良预后有关。IGSF11显著影响人类晚期胶质瘤的预后，可作为胶质瘤的负性预后标志物。IGSF11表达水平与神经胶质瘤患者的总生存期有关，尤其是在与PD-1的协同作用下显示出显著的负性预后效应，提示双重阻断方案的潜在治疗价值^[16]^。肝细胞癌和头颈部鳞癌的预测模型显示出IGSF11具有良好的评估预后的能力，其评分模型有助于区分不同预后的肿瘤患者，为患者的未来管理选择提供新的思路^[19,37,41]^。Nie、Liu等团队^[19,37]^通过风险基因的筛选及构建风险模型确定IGSF11影响肿瘤功能及预后，为区分不同预后情况的口腔鳞状细胞癌患者提供新方案。值得注意的是，在乳腺癌浸润性导管癌中，高表达IGSF11反而预示较好预后情况^[18]^。然而，在子宫内膜癌、皮肤黑色素瘤中，IGSF11表达与其预后无相关性。皮肤黑色素瘤中IGSF11的高水平表达与患者的总生存期和无病生存期无相关性，与临床病理特征及免疫细胞浸润也无相关性^[17]^。IGSF11在所有子宫内膜癌样本中均表达，但与病理特征及预后无关^[25]^，且对其上调的机制尚不清楚，需要进一步的探索研究。这些发现为开发基于IGSF11的精准预后评估系统和靶向治疗策略提供了重要依据。

## 7 小结与展望

IGSF11作为一种新型免疫检查点分子，通过IGSF11/VISTA轴调控T细胞功能，在多种恶性肿瘤中发挥免疫抑制作用。本文系统阐述了IGSF11的结构特征、表达调控机制及其在肿瘤微环境中的功能，并探讨了靶向该通路治疗的药物，为全面理解IGSF11在免疫治疗中的作用及应用前景提供参考。

### 7.1 IGSF11的结构与表达

解析IGSF11的分子结构为靶向药物设计奠定了基础。IGSF11的胞外结构域包含IgV和IgC2结构域，并通过关键残基（如His122、His123）与VISTA特异性结合^[21]^。X射线晶体学和预测模型研究^[30]^进一步揭示了IGSF11/VISTA复合物的空间构象，为开发阻断抗体或小分子抑制剂提供了精确的靶点。

### 7.2 IGSF11在肿瘤调控中的作用

IGSF11在多种实体肿瘤中及部分肿瘤免疫细胞中表达，与其他免疫检查点（如PD-1、CTLA-4）表达也有相关性。在胶质瘤中IGSF11高表达与不良预后显著相关，与PD-1、VISTA表达呈正相关^[16]^；而IGSF11与皮肤黑色素瘤的预后无关，且与PD-1、VISTA表达呈相反关系^[17]^。值得注意的是，在乳腺癌中高表达IGSF11却与较好预后有关^[18]^。IGSF11在肿瘤预后中表现出的特异性，提示了其在肿瘤中通过不同的下游信号通路发挥作用，未来需个体化设计针对各类肿瘤的靶向治疗策略。

IGSF11参与胃癌、头颈部鳞癌增殖、转移、凋亡的调控，但具体机制尚不清楚。IGSF11通过抑制T细胞活化、减少促炎因子（如IFN-γ、IL-17）分泌、促进抗炎因子（如TGF-β、IL-10）产生，塑造免疫抑制微环境，从而促进肿瘤免疫逃逸^[17,27]^。然而，对于IGSF11调控免疫微环境的分子机制尚未完全阐明，尤其是对肿瘤相关巨噬细胞和肿瘤浸润淋巴细胞的功能缺乏系统性研究。此外，IGSF11可能通过调控糖酵解途径（如影响PKM2表达）影响肿瘤代谢^[19]^，但其代谢调控机制仍需深入研究。

### 7.3 靶向IGSF11/VISTA通路的治疗药物

靶向IGSF11/VISTA通路的药物研发已取得初步进展，包括IGSF11靶向药物（如小分子抑制剂K284-3046等）和抗VISTA抗体（如SG7、HMBD-002等）。其中，SG7展现出与VISTA高亲和力及pH非依赖性结合特性，其与PD-1联用在临床前模型中的治疗表现出显著的抗肿瘤效果^[31]^。然而，有关IGSF11靶向药物的临床试验尚未有相关报道，其安全性、药效学及耐药机制仍有待进一步验证。此外，胶质瘤中IGSF11与PD-1协同作用的负性预后效应^[16]^，以及SG7/KVA12123与PD-1/PD-L1联合治疗小鼠肿瘤模型的抗肿瘤效果^[31,40]^，为开发新型联合免疫治疗方案提供了理论依据，表明靶向IGSF11/VISTA轴和PD-1/PD-L1通路的双阻断策略可产生协同抗肿瘤效应，展现出潜在的临床应用前景。

综上所述，IGSF11作为新型免疫检查点分子，在肿瘤免疫调控中扮演重要角色，具有广阔的临床应用前景。然而现有研究主要集中在胶质瘤等肿瘤中，在非小细胞肺癌等实体瘤中仍局限于组学表征阶段。未来仍需深入探索IGSF11在肺癌、食管癌等恶性肿瘤中介导的免疫抑制微环境及代谢重编程调控的相关机制及对肿瘤进展的影响。设计如IGSF11/PD-1等协同阻断联合治疗方案，提高肿瘤治疗应答率，为肿瘤免疫治疗提供更为安全有效的治疗新靶点。
